# Editorial: Identification of immune-related biomarkers for cancer diagnosis based on multi-omics data

**DOI:** 10.3389/fonc.2022.1119622

**Published:** 2022-12-26

**Authors:** Liang Cheng, Xin Zhang, Chuan-Xin Li, Rui Guo, Tianyi Zhao

**Affiliations:** ^1^ Jiangmen Key Laboratory of Clinical Biobanks and Translational Research, College of Bioinformatics Science and Technology, Harbin Medical University, Harbin, China; ^2^ Jiangmen Central Hospital, Jiangmen, China; ^3^ Respiratory Medicine Unit, Department of Medicine & Center for Molecular Medicine, Karolinska Institutet, Stockholm, Sweden; ^4^ Brigham and Women’s Hospital, Harvard Medical School, Stockholm, United States; ^5^ School of Medicine and Health, Harbin Institute of Technology, Harbin, China

**Keywords:** cancer, biomarkers (BMs), multi-omics data, machine learning, cancer diagnosis

Cancer has become one of the leading causes of mortality around the globe. Activating the innate immune signal pathway and inducing the anti-tumor immune response plays a key role in the efficacy of tumor treatment, especially in preventing the recurrence of residual tumor cells. With the development of high-throughput sequencing technology, multi-omics data for cancer has become accessible. These data have given researchers increasing opportunities to explore genetic risk, regulatory mechanisms, and protein function of the immune microenvironment in cancers. However, it is still a big challenge to utilize these data effectively and to mine knowledge from them. Artificial intelligence algorithms and statistical methods have shown great potential to take advantage of omics data and reveal mechanisms of immune function in cancer.

Here, we organized a Research Topic on “*Identification of Immune-Related Biomarkers for Cancer Diagnosis Based on Multi-Omics Data*.” In total, about 30 outstanding works were presented in this thematic issue, ten of which have been highlighted as follows.

- Xu et al. performed a meta-analysis by downloading data from PubMed, Google Scholar, and Embase databases on randomized clinical trials compared ipilimumab, nivolumab, pembrolizumab, or atezolizumab with non-immunotherapy controls. Median overall survival (OS) and median progression-free survival (PFS) were selected to evaluate the efficacy of cytotoxic T-lymphocyte-associated protein 4 (CTLA-4), programmed cell death 1 (PD-1), and programmed death ligand 1 (PD-L1) inhibitors. Utilizing the random-effect model, hazard ratios (HRs) with 95 confidence intervals (CIs) were calculated by R software. The meta-analysis suggested that ICIs were associated with obvious improvements in PFS and OS compared with non-ICI therapies.
- Sun et al. introduced a novel disease-susceptible gene prediction method, XGBG, to study ovarian carcinomas (OCs) in more depth. Firstly, they employed the graph convolutional network (GCN) to reconstruct the gene features based on both gene features and network topological structure. Then, a boosting method was utilized to predict OC susceptible genes. The final XGBG model achieved a high AUC of 0.7541 and an AUPR of 0.8051. This method is helpful in further understanding the etiology and pathology of OC, and may be used as strong theoretical evidence for drug design.
- Chen et al. developed a novel method named “DBN-GTN” to identify gastric cancer-related genes on a large scale. This method built a heterogeneous network using a disease similarity network and a gene interaction network. Meanwhile, the deep belief network (DBN) was applied to reduce the dimension of features. This method used multiple features of genes and gastric cancer to identify the patterns of gastric cancer-related genes, which can be used to find more gastric cancer-related genes, and it performed best among four traditional methods and five similar methods. This paper provides support to further explain the genetic risk, susceptibility, and drug screening of gastric cancer.
- Liu et al. investigated prognostic genes in the tumor microenvironment of colon cancer using gene expression profiles and clinical information from colon adenocarcinoma (COAD) and rectal adenocarcinoma (READ). Meanwhile, they utilized the nine key prognostic genes obtained to build the independent prognostic model. They calculated stromal and immune scores for each sample and identified nine key prognostic genes including *HOXC8, SRPX, CCL22, CD72, IGLON5, SERPING1, PCOLCE2, FABP4*, and *ARL4C* by LASSO Cox regression analysis. This work may help in clinical decisions and improve the prognosis for colon cancer.
- Cao et al. developed an accurate and interpretable attention-based hybrid approach, DeepARC, which combined a convolutional neural network (CNN) and recurrent neural network (RNN) to predict transcription factor binding sites (TFBS). Taking advantage of the attention mechanism, DeepARC can gain greater access to valuable information about the motif and bring interpretability to the work of searching for motifs through the attention weight graph. Moreover, DeepARC achieved an average area under the receiver operating characteristic curve (AUC) score of 0.908 on five cell lines in the benchmark dataset. This method predicts better than existing state-of-the-art methods and has good interpretability.
- Qiu et al. developed a novel deep-learning framework to study the association between MSI status and several molecules including mRNA, miRNA, lncRNA, DNA methylation, and copy number variation (CNV) using colorectal cancer data from The Cancer Genome Atlas (TCGA). The fusion models integrating the H&E image with a single type of molecule has higher prediction accuracies than that using the H&E image alone, with the highest AUC of 0.952 achieved when combining the H&E image with DNA methylation data. This study may have clinical significance in practice and provide a reference for future studies.
- Duan et al. proposed an SVM-based method, SVMMDR, to predict the relationship between miRNAs and drug resistance based on the miRNAs-drug resistance association data from the ncDR database. The SVMMDR integrated miRNAs-drug resistance association, miRNAs sequence similarity, drug chemical structure similarity, and other similarities, extracted path-based Hetesim features, and obtained inclined diffusion features through restart random walk. By combining the multiple features, the prediction score between miRNAs and drug resistance was obtained based on the SVM. The final the average AUC of the SVMMDR method was 0.978 in 10-fold cross-validation. This work shows that SVMMDR has a significant performance advantage compared with existing methods.
- He et al. proposed a method for large-scale identification of esophageal cancer-related genes by computational methods, GCNLMF, to improve the efficiency of esophageal cancer genetic susceptibility research. This method fused graph convolutional networks and logical matrix factorization to effectively identify esophageal cancer-related genes through the association between genes. The GCNLMF achieved an AUC of 0.927 and AUPR of 0.86 in 10-fold cross-validation. In the final comparison with the other five methods, GCNLMF performed best. This study provides a new algorithm for finding signature genes in esophageal cancer and offers new insights into the future development of esophageal cancer research.
- Li et al. first downloaded the mRNA, microRNA (miRNA), long non-coding RNA (lncRNA), copy number variation (CNV) data, and clinical information of patients with endometrial cancer(EC) from The Cancer Genome Atlas (TCGA). Then, differential expression analyses were performed to screen potential prognostic markers and establish prediction models using three classifiers. Finally, the prediction model achieved an area under the curve of 0.763, and an accuracy of 0.819 under 10-fold cross-validation. This work develops a computational model using omics information, which can predict the recurrence and metastasis risk of EC accurately, thereby avoiding improper treatment, and improving the prognosis of patients.
- Li et al. proposed a novel deep-learning method named Deep-LC for predicting NSCLC-related genes. Firstly, they built a gene interaction network and used graph convolutional networks to extract features of genes and interactions between gene pairs. Then, a simple convolutional neural network module was used as the decoder to decide whether the gene was related to the disease. Deep-LC is an end-to-end method, and from the evaluation results, they can conclude that Deep-LC performs well in mining potential Non-Small Cell Lung Cancer-related genes and performs better than existing state-of-the-art methods. This work provides new insights for future research in non-small cell lung cancer.

## Author contributions

All authors listed have made a substantial, direct, and intellectual contribution to the work and approved it for publication.

